# PROTAC-Splitter: a machine learning framework for automated identification of PROTAC substructures

**DOI:** 10.1186/s13321-025-01135-9

**Published:** 2026-02-20

**Authors:** Stefano Ribes, Ranxuan Zhang, Télio Cropsal, Anders Källberg, Christian Tyrchan, Eva Nittinger, Rocío Mercado

**Affiliations:** 1 Department of Computer Science and Engineering, Chalmers University of Technology and University of Gothenburg, Chalmersplatsen 1, 412 96 Gothenburg, Sweden; 2 Medicinal Chemistry, Discovery Sciences, BioPharmaceuticals R&D, AstraZeneca, Pepparedsleden 1, 431 83 Gothenburg, Sweden

**Keywords:** Drug discovery, Machine learning, Targeted protein degradation, PROTAC, Cheminformatics

## Abstract

Proteolysis-targeting chimeras (PROTACs) are heterobifunctional molecules composed of an E3 ligase ligand, a linker, and a warhead targeting a protein of interest. Despite their modular structure, accurately identifying and annotating these components in PROTACs is challenging and typically relies on manual curation and predefined substructure matching. To address this, we developed PROTAC-Splitter, a machine learning framework designed for automated annotation of PROTAC substructures. To address data scarcity, we generated and openly released a synthetic dataset containing approximately 1.3 million PROTAC structures with annotated ligand splits. Leveraging this dataset, we developed two complementary approaches for PROTAC substructure annotation: a Transformer-based sequence-to-sequence model and a graph-based XGBoost model. We evaluated both approaches on held-out public data and structurally novel PROTACs from AstraZeneca’s proprietary collection. The Transformer-based model achieved high exact-match accuracy (86%) on public data but dropped significantly (18%) on structurally novel internal PROTACs due to occasional hallucinations. In contrast, the XGBoost model can ensure chemical validity and perfect reassembly accuracy on both sets, with lower exact-match accuracy on open-data (42.2%) but comparable performance on the internal set (23%). To improve reliability, we implemented a wrapper function for the Transformer (Transformer-$\Delta$), which corrects partial prediction errors, raising reassembly accuracy to 96% on public and 70% on internal datasets. Combining the strengths of both models, we propose a hybrid approach that reliably annotates PROTACs across diverse chemical spaces. PROTAC-Splitter provides a robust, scalable tool to facilitate automated PROTAC analysis and is available open-source at https://github.com/ribesstefano/PROTAC-Splitter

**Supplementary Information:**

The online version contains supplementary material available at 10.1186/s13321-025-01135-9.

## Scientific contribution

We address the challenging problem of automated PROTAC annotation by releasing an openly accessible, chemically realistic synthetic dataset of approximately 1.3 million PROTAC-like molecules with expertly annotated ligand, linker, and warhead substructures. Leveraging this resource, we introduce PROTAC-Splitter, a hybrid machine learning framework combining a Transformer-based sequence-to-sequence model and a chemically constrained XGBoost graph model, both readily adaptable through fine-tuning on proprietary datasets. Extensive benchmarks on public and AstraZeneca-internal compounds demonstrate that our hybrid approach achieves state-of-the-art reassembly accuracy (up to 96%) while ensuring interpretability, chemical validity, and robustness across diverse chemical spaces.

**Supplementary Information:**

The online version contains supplementary material available at 10.1186/s13321-025-01135-9.

## Introduction

Proteolysis-targeting chimeras (PROTACs) are heterobifunctional molecules that recruit an E3 ubiquitin ligase to a protein of interest (POI), triggering its degradation by the proteasome rather than inhibition[1]. Each PROTAC comprises three components: an E3 ligase ligand, a warhead that binds the POI, and a linker that tethers the two. Small changes to any component can markedly alter their degradation efficiency and pharmacokinetics[2, 3], such that the rational design of PROTACs necessitates careful optimization of each component.

Despite their apparent modularity, reliably decomposing PROTAC structures into their constituent ligands is far from trivial. Current workflows depend on manual curation or rigid substructure matching and quickly break down as datasets grow; variations in attachment points, ring fusions, and stereochemistry exacerbate the difficulty. Furthermore, these types of annotations are frequently missing from existing structured data sources for PROTACs[4, 5]. As a result, what might appear to be a simple parsing task quickly becomes a complex problem requiring automated, data-driven solutions.

To overcome this challenge, we introduce PROTAC-Splitter, a machine learning (ML) framework that automatically extracts the E3 ligase ligand, linker, and warhead from a PROTAC given an input string representation using SMILES. Because only $\sim$5,000 publicly available PROTACs are available in the public domain, and even fewer annotated, as part of this work we also generated and released a synthetic set of 1.3 million PROTAC-like molecules with realistic attachment-point chemistry. Models trained on this corpus were evaluated on real, held-out public datasets and on structurally novel degraders from the AstraZeneca proprietary collection, demonstrating robust performance across both in- and out-of-distribution (OOD) chemistry.

The PROTAC-Splitter presents two complementary approaches: (1) a Transformer sequence-to-sequence model, which attains 86% exact-match accuracy and 96% reassembly accuracy on a held-out public test set; and (2) a lightweight XGBoost model which guarantees 100% chemical validity and perfect reassembly on every dataset, albeit with lower exact-match accuracy (42% public, 23% internal). To improve reliability, we implemented a wrapper function to fix poor predictions by the Transformer model (Transformer-$\Delta$); this fixing function can correct partial prediction errors, raising reassembly accuracy to 96% on public and 70% on internal datasets. We propose that, for researchers aiming to apply the PROTAC-Splitter in far OOD settings, a hybrid strategy of accepting Transformer predictions that pass validity checks and defaulting to XGBoost otherwise can deliver the best of both worlds, yielding correct, fully reassembling splits for 100% of the data while retaining a high match rate on in-distribution molecules.

In this work, we make the following key contributions. First, we offer a large-scale synthetic PROTAC dataset of 1.3M structures complete with carefully curated and chemically realistic ligand annotations, available open-source to facilitate further research and method development. Second, we present two complementary splitting models, (1) a Transformer-based sequence-to-sequence model and (2) an XGBoost-based graph model, both of which are accessible, ready-to-use, and readily adaptable through fine-tuning on private datasets. Last, we conduct extensive benchmarking covering both public and proprietary data, highlighting both the in-distribution and OOD performance of our models. Our results demonstrate the potential for robust, accurate splitting of PROTAC molecules, laying a solid foundation for further advancements in automated PROTAC analysis and targeted protein degrader design. All code for this work is available at github.com/ribesstefano/PROTAC-Splitter, and all data, including the synthetic PROTAC dataset and pretrained models, is available on Zenodo at doi.org/10.5281/zenodo.15797310.

### Background

Recent years have seen a rapid growth of ML approaches for small-molecule design, and targeted protein degraders have now entered that stream. General-purpose molecular language models such as Chemformer [6] and ChemBERTa-2 [7] laid the groundwork by demonstrating that Transformer architectures can capture chemical syntax and reaction semantics directly from SMILES. Building on these advances, several groups have adapted sequence-to-sequence or fragment-linking paradigms specifically to PROTACs. SyntaLinker generates linkers between fixed warhead and E3-ligase fragments and was one of the first works to show that Transformer cross-attention can reproduce known PROTAC linkers [8]. PROTAC-RL couples instead a graph Transformer with reinforcement learning (RL) to optimize not only connectivity but also ternary-complex formation, reportedly outperforming physics-based docking on benchmark degraders [9]. Also using RL, DRLinker introduced a dual-representation scheme that fuses SMILES and molecular graphs, enabling one-shot generation of full PROTACs with chemically plausible attachment points [10]. A comprehensive survey by Gharbi and Mercado [2] cataloges these and other emerging methods, yet also highlights persistent bottlenecks: public data comprise merely $\sim$5K unique PROTACs, and most public structures lack explicit annotations for the warhead, linker, and E3 ligase binders components. This scarcity hampers ML model development in the PROTAC space. We close that gap by releasing a million-scale synthetic dataset with atom-level splits and by showing, through extensive benchmarking on real public and proprietary PROTACs, that models trained on this synthetic corpus generalize meaningfully to real-life chemistry.

Beyond structure generation, ML has also begun to tackle downstream property-prediction tasks. For example, Ribes et al. [11] showed that shallow deep neural networks can predict degradation efficiency directly from PROTAC structures and precomputed protein embeddings (ROC-AUC $>0.80$ on PROTAC-DB data). Similarly, DeepPROTACs[12] applies graph convolution networks to ligand–pocket pairs with a BiLSTM linker encoder, achieving 77.95% accuracy on degradation prediction for similar public PROTAC structures. More recently, PROTAC-STAN[13] and DegradeMaster[14] enhance model interpretability and performance on degradation activity prediction by incorporating structural information via a ternary attention mechanism in PROTAC-STAN and an E(3)-equivariant graph neural network (GNN) in DegradeMaster. Integrating such property predictors with generative splitters, such as the one introduced here, could streamline the design–make–test loop for PROTACs by enabling more fine-grained control over predictions based on what substructures a PROTAC comprises of.

## Methods

**Fig. 1:**
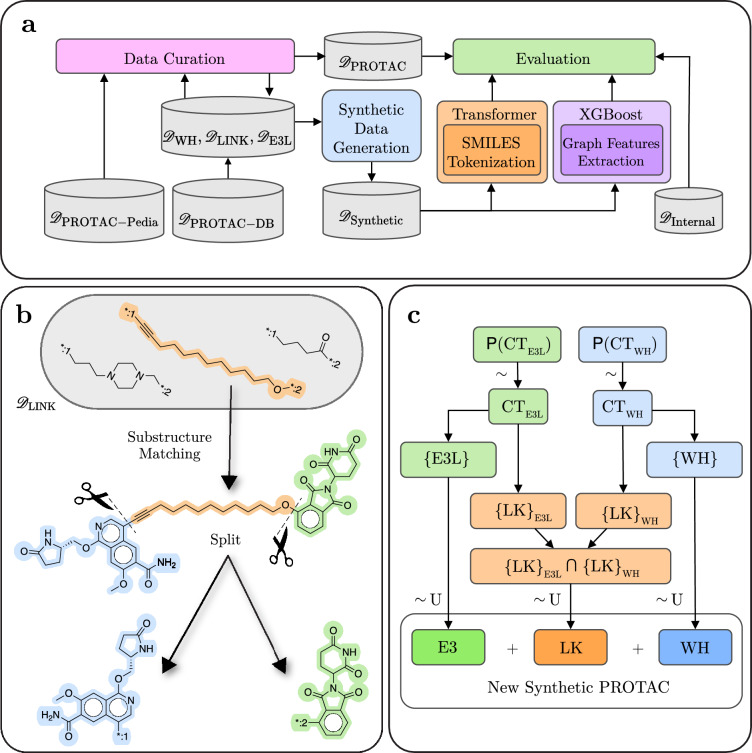
(**a**) Summary of the data curation, model training, and evaluation pipeline developed in this work. (**b**) Illustration of the linker matching procedure used in the dictionary-based substructure annotation procedure (Section 2.2). (**c**) High-level overview of the synthetic data generation process, designed to reflect the distribution of connection types between the substructures in the public PROTACs curated from PROTAC-DB and PROTAC-Pedia. Here, $\textsf{P}$ denotes a probability distribution of connection types, and $\sim$ denotes sampling from a distribution. Additional details on sampling substructures can be found in Appendix C.3.

### Data

Figure 1 summarizes our data processing and model development pipeline.

#### Data sources

*Public data*

We collected PROTAC structures from two open-source datasets: PROTAC-DB 3.0 (accessed November 2024) [15] and PROTAC-Pedia [4]. After SMILES canonization, PROTAC-DB includes 3,270 unique PROTACs, while PROTAC-Pedia adds 1,178 more. Although none of the PROTACs are systematically annotated with their corresponding substructures in the downloadable data, both data bases provide sets of substructures: we extracted 569 and 183 warheads, 2,490 and 664 linkers, and 107 and 69 E3 ligase ligands from PROTAC-DB and PROTAC-Pedia, respectively. Notably, only PROTAC-DB includes attachment point information. We found these to be occasionally chemically inconsistent; for instance, splits sometimes ignore obvious cleavage points like amide bonds. As such, we developed an algorithm for systematic substructure annotation that was part of the extensive data curation process applied to the public data (Section 2.2).

*Internal data*

**Fig. 2:**
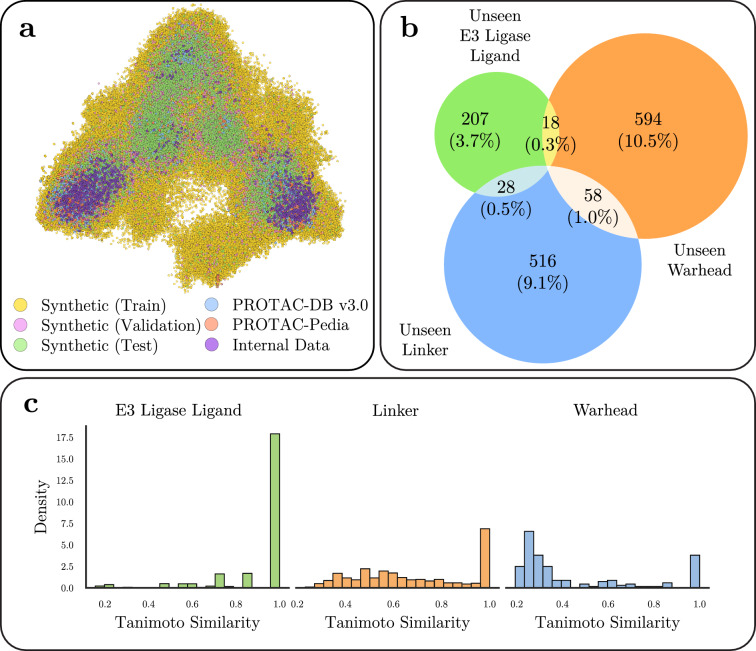
(**a**) Principal component analysis (PCA) visualization of the chemical space covered by the synthetic PROTACs constructed herein (yellow, pink, and green), the curated public PROTACs (blue and red), and PROTACs from AstraZeneca’s internal data collection (purple). PCA was performed on 512-bit Morgan fingerprints (radius = 2). (**b**) Venn diagram illustrating the number of PROTACs in the public (real) test set that have one, two, or three substructures not seen in the synthetic train set. The percentage reports the fraction of used substructures within each substructure class. (**c**) Histograms of the Tanimoto similarity between substructures in the internal test set and substructures in the synthetic train set (from left to right: E3 ligase ligands, linkers, warheads). Histograms show the bin count corresponding to the highest Tanimoto similarity for each compound, using 512-bit Morgan fingerprints (radius = 2), in the internal data to the compounds in the synthetic training set.

 As an external test set, we tested the model on a dataset of 2,256 PROTACs from AstraZeneca’s proprietary data collection. The overlap and similarity of this internal data with the other datasets used in this work is illustrated in Figure 2.

#### Data curation and standardization

After canonicalization, we merged the public datasets and removed any duplicates of PROTACs and their ligands from PROTAC-DB and PROTAC-Pedia using exact string matching (SMILES comparisons). Then, we created a set of four SMILES dictionaries, one each for full PROTACs ($\mathscr {D}_{\textrm{PROTAC}}$), E3 ligase binders ($\mathscr {D}_{\textrm{E3L}}$), linkers ($\mathscr {D}_{\textrm{LINK}}$), and warheads ($\mathscr {D}_{\textrm{WH}}$). Any fragmented ligands or PROTACs, indicated with a dot “.” in SMILES notation, were discarded to ensure molecular integrity. When adding any PROTAC or substructure to a dictionary, we also include a copy of it with removed stereochemistry information.

### Dictionary-based substructure annotation

Due to inconsistent annotations in public data, we developed an algorithmic approach to infer the constituent substructures of a PROTAC by referencing the sets of available ligand dictionaries. The goal was to assign a warhead, linker, and E3 ligase ligand to each of the 5,670 PROTACs collected from PROTAC-DB and PROTAC-Pedia, each with their corresponding attachment points, in this way generating the “computationally derived” substructure labels for what would become the public, held-out test set. In addition to creating substructure annotations, the algorithm led to the extraction of additional ligands and linkers than those originally reported in PROTAC-DB and PROTAC-Pedia; the approach is described below.

*Inferring substructures*

The core idea behind substructure inference is that, if two known ligands (*e.g.*, warhead and E3 ligase binder) can be matched as substructures within a PROTAC, the third component (typically the linker) can be inferred by subtracting the matched atoms from the parent molecule. An example of substructure matching is shown in Figure 1b.

We first attempt to identify both the warhead and E3 ligase binder by matching entries from $\mathscr {D}_{\textrm{WH}}$ and $\mathscr {D}_{\textrm{E3L}}$ as substructures of the input PROTAC. If both matches are found, the linker is inferred by removing their atoms from the full molecule. If only one match is found, we search the linker dictionary $\mathscr {D}_{\textrm{LINK}}$, starting from the largest candidates, for a match that enables recovery of the remaining ligand. In more difficult cases (*e.g.*, when neither warhead nor E3 binder matches) we fall back to matching linkers alone, starting the substructure search from the largest linker. The remaining two substructures are then extracted and labeled as warhead or E3 binder based on their Tanimoto similarity to known entries in $\mathscr {D}_{\textrm{WH}}$ and $\mathscr {D}_{\textrm{E3L}}$. Note that we prioritize ligand matches (warhead and E3 ligand) first, falling back to linker-only matching when necessary.

All inferred splits are validated via reassembly: we confirm that the three predicted components reconstruct the original PROTAC exactly, without missing or extraneous atoms. To further improve chemical plausibility, we apply a post-processing step that adjusts attachment points when amide or ester groups are located within one bond of the cleavage site. In such cases, the attachment point is shifted to reflect more realistic synthetic strategies.

*Iterative refinement*

To improve coverage, the substructure matching algorithm was embedded in an iterative loop. After each round of matching, newly identified ligands were added to the corresponding dictionaries ($\mathscr {D}_{\textrm{WH}}$, $\mathscr {D}_{\textrm{LINK}}$, $\mathscr {D}_{\textrm{E3L}}$), and the process was repeated until convergence. Using this approach we were able to successfully annotate 5,562 (98.8%) of PROTACs; the remaining 103 (1.8%) structures were manually annotated, with emphasis on selecting chemically plausible splits (see Appendix B).

In total, our curation pipeline yielded 5,670 PROTACs with full substructure assignments. The final dictionaries contain 253 unique E3 ligase binders ($\mathscr {D}_{\textrm{E3L}}$), 1,926 unique linkers ($\mathscr {D}_{\textrm{LINK}}$), and 885 unique warheads ($\mathscr {D}_{\textrm{WH}}$), each with clearly defined attachment points. Attachment sites were encoded using dummy atoms: [:1] for the warhead-facing end and [:2] for the E3-facing end. Linker SMILES include both attachment points, while warhead and E3 ligand SMILES each contain only one.

### Synthetic dataset generation

Although dictionary-based splitting yields a high-quality dataset of 5,670 PROTACs and their substructure labels, its size is limited in the context of language models, which typically require significantly larger scales of training data. This motivated the construction of a *synthetic* PROTAC dataset, formed by recombining the curated ligand components and thus enabling broader chemical space coverage for training a language model.

To this end, we systematically recombined ligands from $\mathscr {D}_{\textrm{E3L}}$, $\mathscr {D}_{\textrm{LINK}}$, and $\mathscr {D}_{\textrm{WH}}$, all annotated with attachment points, to generate new PROTACs. Using the ligand sampling strategy detailed in Appendix C.3, designed to respect the distribution of bond types at the attachment points, we produced 1,298,401 training examples, along with 11,330 and 11,308 molecules for validation and testing, respectively. The original 5,670 curated PROTACs, which correspond to real and experimentally-verified structures, were reserved as a held-out test set. Rather than exhaustively enumerating all possible ligand combinations, we chose to cap generation at $\sim$1 million PROTACs for practical reasons.

To simulate real-world generalization scenarios where unseen or proprietary ligands may appear, we intentionally excluded certain ligands from the training set. Specifically, we clustered ligands using Taylor-Butina clustering on 1,024-bit Morgan fingerprints (radius = 16, with chirality), and assigned PROTACs containing ligands from select clusters to validation and test splits. This ensured that a fraction of PROTACs in these splits contain at least one ligand not encountered during training (Figure 2b), and is also the reason for the “uneven” numbers of structures in the training, validation, and testing splits. Full dataset statistics and overlap metrics are presented in Table 1. More information on clustering the synthetic data is reported in Appendix C.2. Appendix C.1 includes an analysis on the selected Morgan fingerprint parameters.

### Two PROTAC-Splitter model paradigms

To automate the decomposition of PROTAC molecules into their three constituent substructures, we developed two complementary ML models: one based on XGBoost [16], and the other on a Transformer architecture [17]. These models differ in representation and learning paradigms—one operating on graph-derived features, the other on SMILES sequences—but both share the goal of accurate splitting points for a given PROTAC. An overview of the models is presented in Figure 1a.

#### XGBoost-PROTAC-Splitter

The XGBoost-PROTAC-Splitter uses graph-derived and chemical features to identify which chemical bonds in a PROTAC molecule correspond to splitting points between its substructures. We formulate this as a binary classification task, where each bond is scored by an XGBoost-based model. The top two predicted bonds are then used to fragment the PROTAC into three components using RDKit.

Once split, we identify the linker as the fragment containing two attachment points. Then, to distinguish between the E3 ligase ligand and the warhead, we compute the average Tanimoto similarity of the remaining two fragments against a reference set of 50 representative E3 ligase ligands, selected via k-means clustering from the training set. The fragment most similar to this reference set is labeled as the E3 binder; the other is assigned as the warhead. Finally, we apply the same attachment point refinements as described in Section 2.2 to ensure chemically reasonable connections (*i.e.*, connecting ligands at amide or ester bonds).

*Feature extraction*

Each PROTAC is represented as a molecular graph and a corresponding line graph, *i.e.*, a graph were nodes correspond to the edges of the original graph and edges nodes. For each bridge node in the line graph (*i.e.*, bonds that would disconnect the graph if removed), we compute a normalized betweenness centrality score [18] using networkx. For each bond, we also compute the first-, second-, and third-order degrees of its atoms, both in the original and line graph representations, and we include the following basic chemical features: bond type (single, double, or triple) and the element types of the connected atoms. To avoid ambiguous cuts, we restrict classification to bridge bonds (non-ring). Finally, we compute 512-bit Morgan fingerprints (radius = 6) for the entire PROTAC and include them as global molecular descriptors.

*Model pipeline and training*

We wrap the XGBoost classifier into a scikit-learn pipeline comprising: (1) a truncated singular value decomposition (SVD) module that reduces the molecular fingerprint to 50 dimensions; (2) one-hot encoding of categorical bond-level features; and (3) a SMOTE oversampler [19] to address class imbalance, as only two bonds per PROTAC (out of on average 40) are “positive” labels, *i.e.*, connection points.

The model was trained on 1% of the synthetic training set (randomly sampled) and evaluated on 50% of both the validation and testing data, yielding 12,984, 5,665, and 5,654 PROTACs per split, respectively. This corresponds to 515,260, 227,058, and 227,350 bond-level data points, respectively. No hyperparameter optimization was conducted, as the model performed robustly with initial hand-tuned settings.

#### Transformer-PROTAC-Splitter

The Transformer-PROTAC-Splitter formulates the substructure-splitting task as a sequence-to-sequence translation problem. The model follows an encoder-decoder architecture, where the encoder generates contextual embeddings of the input PROTAC SMILES, and the decoder autoregressively outputs the corresponding substructure SMILES. The encoder is initialized from a pretrained ChemBERTa-v1 model [7], while the decoder is a modified ChemBERTa-v1 variant augmented with cross-attention layers and a linear output head for token prediction. During training, only the cross-attention layers and the output head are randomly initialized and trained from scratch; the remaining weights are fine-tuned from the pretrained checkpoints.

We represent PROTACs and their constituents using SMILES strings. The model takes a canonicalized PROTAC SMILES as input and outputs a concatenated, dot-separated SMILES string representing the E3 binder, linker, and warhead, in that order. To improve generalization, we apply SMILES randomization to 15% of the training inputs, leaving the output (*i.e.*, target substructure SMILES) unchanged. At inference, we use beam search decoding with a width of $k=5$ during sampling.

*Transformer-* $$\Delta$$ *Model*

Autoregressive generation can lead to minor structural errors, such as extra or missing atoms, particularly in long, repetitive linkers (*e.g.*, an extra “C” in a linear alkyl chain like “CCCC...”). In the context of language models, this behavior is better known as *hallucination*. Nevertheless, because the original PROTAC SMILES is known, and any two correctly predicted components uniquely determine the third, such errors can often be corrected, provided the errors are not great nor present on all substructures

To enable this correction, we introduce a deterministic wrapper around the original Transformer model, which we refer to as Transformer-$\Delta$. This fixing function identifies the erroneous component by comparing the generated fragments to the input PROTAC, and infers the correct ligand by subtracting the accurate fragments from the parent molecule, mirroring the logic used in our data curation pipeline. For implementation details, see Appendix A.

### Evaluation metrics

We evaluated model performance using a suite of metrics tailored to our SMILES generation task:

- *Validity*: Whether the generated SMILES string can be successfully parsed by RDKit.
- *Exact-match accuracy* (or *match rate*): The proportion of predictions that exactly match the computationally derived labels, including correct placement of attachment points.
- *Reassembly accuracy*: Whether the three predicted substructures, when joined together and canonicalized, reproduce the original input PROTAC SMILES exactly.
- *Extra atoms*: The difference in heavy atom count between the input PROTAC and the reassembled prediction. Positive values indicate missing atoms; negative values reflect hallucinated (extraneous) atoms.

For practitioners, each metric offers a distinct lens on model utility: exact-match and reassembly accuracy reflect how reliably the model produces chemically valid and synthetically useful splits, while validity ensures that outputs are parsable for downstream cheminformatics workflows. The extra atoms metric is particularly important for quantifying subtle structural errors or hallucinations. For the Transformer model, which uses beam search ($k=5$) for generation, we report all metrics based on the top-1 (highest-likelihood) prediction. Top-5 results and the evaluation procedure are detailed in Appendix D.5. The effect of applying SMILES augmentation on performance is instead reported in Appendix D.3.

## Results

### The synthetic dataset captures real PROTAC space

**Table 1:** Comparison of substructures present in the synthetic PROTAC dataset to the public and internal PROTAC data

Dataset	Size	Unique WHs	Unique Linkers	Unique E3s
Public (Hold-out Test)	5,670	885	1,926	253
Internal (Hold-out Test)	2,256	171	590	119
Synthetic (Train)	1,297,604	745	1,626	217
Synthetic (Validation)	11,315	883	1,925	253
Synthetic (Test)	11,296	883	1,925	253

Note that not all the known ligands from the public dataset were used to create the synthetic dataset: $\mathscr {D}_{\textrm{WH}}$=885 unique warheads; $\mathscr {D}_{\textrm{LINK}}$=1,926 unique linkers; and $\mathscr {D}_{\textrm{E3L}}$=253 unique E3 ligase binders.

To enable robust training of PROTAC-splitting models, we constructed a large-scale synthetic dataset by recombining curated ligand components. This synthetic set covers similar chemical space as real PROTACs while introducing controlled substructure novelty to enable us to better test the generalization capabilities of our models. Each synthetic PROTAC is annotated with a triplet of ligands (E3 ligase binder, linker, and warhead), generated via semi-random sampling of component dictionaries ($\mathscr {D}_{\textrm{E3L}}$, $\mathscr {D}_{\textrm{LINK}}$, and $\mathscr {D}_{\textrm{WH}}$) while preserving realistic bond-type distributions at attachment points.

Table 1 summarizes key statistics of the synthetic dataset, and quantifies the diversity of the chemical space encapsulated by the enumerated structures. The overlap and similarity of the synthetic data with the other datasets used in this work is illustrated in Figure 2a; here, principal component analysis (PCA) of molecular fingerprints reveals that synthetic PROTACs span a similar chemical space as real PROTACs from PROTAC-DB and PROTAC-Pedia. Furthermore, the training, validation, and test splits appear well-distributed across this space.

To assess novelty, Figure 2b quantifies the presence of “unseen” substructures (*i.e.*, ligands not present in the training set) within the held-out test set. By design, the held-out test set includes compounds outside the training distribution: 23.3% of held-out test PROTACs contain at least one unseen component (warhead, linker, or E3 ligand), and 1.8% contain at least two unseen components. Notably, no test-time molecules contain three unseen substructures simultaneously, reflecting our strategy of balancing the task’s challenge with the scenarios likely to be encountered in real-world settings.

### PROTAC-Splitter models achieve high accuracy on public data

**Table 2:** Top-1 evaluation metrics on the public and internal datasets for both the XGBoost- and Transformer-based PROTAC-Splitter models

Dataset (Size)	Metric	Heuristic	XGBoost	Transformer	Transformer-$\Delta$
Public (5,670)	Validity	99.15%	**100%**	97.48%	99.51%
	Reassembly	98.78%	**100%**	79.39%	96.28%
	Exact-match	14.45%	42.20%	73.45%	**85.96%**
	Extra atoms	0.42	**0**	2.06	0.46
Internal (2,256)	Validity	-	**100%**	73.49%	88.92%
	Reassembly	-	**100%**	9.75%	70.35%
	Exact-match	-	**22.96%**	4.26%	18.71%
	Extra atoms	-	**0**	17.99	7.70

The “Transformer-$\Delta$” column reports Transformer performance after applying the fixing wrapper function. For all metrics except *Extra Atoms*, a higher number indicates better performance. Note that the extra atoms are only computed for the predictions that fail reassembly.

We benchmarked both models on the 5,670 PROTACs in the held-out open-source dataset, comprising real, curated molecules from PROTAC-DB and PROTAC-Pedia. All four evaluation metrics (validity, reassembly accuracy, heavy atom difference, and exact-match accuracy) were computed. As shown in Table 2, the XGBoost-PROTAC-Splitter achieves perfect scores for validity and reassembly accuracy, reliably producing chemically coherent splits with no hallucinated atoms. However, since it selects cut sites based primarily on graph-based features, it attains a relatively low exact-match accuracy of 42.20%.

In contrast, the Transformer-based models—especially Transformer-$\Delta$, which applies a fixing function to correct minor generation errors—achieve much higher exact-match accuracy, with Transformer-$\Delta$ reaching 85.96%. This indicates a much stronger alignment with human-annotated and computationally-derived labels, which may reflect synthetic accessibility or experimentally validated cut sites.

If exact label matching is not essential, Transformer-$\Delta$ also achieves 96.29% reassembly accuracy, indicating strong functional correctness even for slightly shifted cut points. In practice, this suggests a useful hybrid strategy: use Transformer-$\Delta$ to split most PROTACs to maximize accuracy, and fall back to the XGBoost model for rare edge cases that fail reassembly, thereby ensuring robust, domain-complete predictions.

We also compare our models to a learning-free heuristic algorithm, detailed in Appendix E. Despite showing promising results, AI-based splitting models outperform the algorithm on the held-out open set, particularly on the exact-match score, as reported in Table 2.

### PROTAC-Splitter models maintain good performance on internal data

**Table 3:** Top-1 evaluation metrics, broken down by substructure class, on the public and internal datasets for both the XGBoost- and Transformer-based PROTAC-Splitter models

Dataset (Size)	Substructure	Metric	XGBoost	Transformer	Transformer-$\Delta$
Public (5,670)	Warhead	Validity	**100%**	97.97%	99.59%
		Exact-match	49.31%	85.71%	**90.97%**
		Extra atoms	1.56	0.46	**0.04**
	Linker	Validity	**100%**	99.79%	99.95%
		Exact-match	43.01%	83.01%	**87.91%**
		Extra atoms	1.58	0.25	**0.07**
	E3 Ligand	Validity	**100%**	99.70%	99.95%
		Exact-match	72.05%	89.41%	**91.71%**
		Extra atoms	3.14	0.05	**0.04**
Internal (2,256)	Warhead	Validity	**100%**	73.98%	89.36%
		Exact-match	**40.43%**	11.92%	36.61%
		Extra atoms	**2.45**	7.40	3.60
	Linker	Validity	**100%**	98.89%	98.94%
		Exact-match	**43.01%**	23.05%	18.97%
		Extra atoms	**0.58**	-0.74	-1.00
	E3 Ligand	Validity	**100%**	99.20%	99.20%
		Exact-match	40.16%	40.20%	**42.86%**
		Extra atoms	-3.03	0.84	**0.78**

The “Transformer-$\Delta$” column reports Transformer performance after applying the fixing wrapper function. For all metrics except *Extra Atoms*, a higher number indicates better performance. Note that the extra atoms are only computed for the predictions that fail reassembly.

To evaluate real-world generalization, we tested the models on 2,256 compounds from AstraZeneca’s proprietary PROTAC collection. These compounds, as shown in Figure 2c, are generally dissimilar to structures in the public data (real PROTACs from PROTAC-DB and PROTAC-Pedia), especially when it comes to the warheads. Evaluating the PROTAC-Splitter models on these compounds can thus provide insight into how well the models extrapolate to novel chemical scaffolds.

As shown in Table 3, the XGBoost model maintains perfect validity (100%) across all substructures, even on internal data. This is by design: its rule-based, non-generative nature prevents structural hallucinations, ensuring chemically valid and reassembling outputs. However, this reliability comes at the cost of flexibility, as exact-match accuracy remains modest ($\sim$23%) due to its inability to model more nuanced, dataset-specific splitting patterns. In contrast, the Transformer model (without the fixing function) struggles on these OOD compounds, particularly for warheads, where validity drops to 74% and exact-match accuracy to just 12%. Applying the fixing function in the Transformer-$\Delta$ variant substantially improves both validity (to 89.4%) and exact-match performance for warheads (to 36.6%), while reducing structural errors across all components. Notably, E3 ligands, which tend to be more conserved and recognizable, are recovered at a relatively strong 42.9% exact-match accuracy by the Transformer-$\Delta$ model.

Importantly, if exact label matching is not required, Transformer-$\Delta$ achieves a 70.35% reassembly accuracy on the internal test set. This is an encouraging result given the novelty of these PROTACs relative to public data. These results emphasize the value of the fixing function in correcting minor structural errors and recovering meaningful substructures, especially when deploying the model on previously unseen chemistries. Further, they highlight a clear complementary to both models: XGBoost offers robustness and structural integrity, while Transformer-$\Delta$ provides more accurate and flexible splits when minor errors can be corrected.

## Discussion

### Scalable learning from synthetic data, up to a point

The synthetic dataset of approximately 1.3M PROTACs used to train PROTAC-Splitter captures a substantial portion of the chemical diversity achievable from the curated ligand dictionaries (253 E3 ligase binders, 1,927 linkers, and 883 warheads). The choice to limit the synthetic dataset size was practical, balancing computational tractability with sufficient coverage of the chemical space. Data efficiency analysis (Appendix D.2) highlights diminishing returns: increasing training data from 25% to 50% improves reassembly accuracy by 0.72 percentage points, whereas expanding from 75% to 100% yields only a minor 0.54-point increase. This suggests that the current dataset size captures the majority of learnable structure-splitting patterns and that further gains may require incorporating novel chemotypes not present in the existing ligand pool.

### Generalization to unseen substructures is critical for real-world applicability

Real-world PROTACs, especially those from proprietary sources, often include substructures that differ significantly from those found in public datasets[20]. This mismatch is particularly evident for warheads here. Evaluation of PROTAC-Splitter on AstraZeneca’s internal data highlights the challenge of generalizing to such OOD chemistry. Compared to performance on public data, the Transformer-$\Delta$ model exhibits substantial declines: match rate drops by 67.25% and reassembly accuracy by 25.93%. A more granular analysis (Table 3) reveals that most of this performance loss stems from the warhead component, which is structurally more diverse and less represented in public sources. This is corroborated by Figure 2c, which shows that the majority of internal warheads have a Tanimoto similarity below 0.5 relative to public training examples.

To close the performance gap on OOD data, domain adaptation strategies are crucial. Fine-tuning the Transformer-$\Delta$ model on a small, representative set of internal PROTACs, or applying transfer learning to better align it with proprietary chemical space, could substantially improve generalization. In parallel, updating the ligand dictionaries or retraining the XGBoost classifier with internal structures may enhance its splitting precision. However, fine-tuning a model is not always desirable. Therefore, an alternative hybrid inference strategy can be adopted that combines the best of both models without additional training. In this setup, predictions are first generated using Transformer-$\Delta$ with beam-search decoding. If the predictions pass both validity and reassembly checks, they are retained. If not (*e.g.*, approximately 4% of cases in public data and up to 30% in internal data) the system falls back to XGBoost, which guarantees structurally valid outputs by design. This hybrid approach leverages the high match rate of the Transformer-$\Delta$ on in-distribution inputs while relying on XGBoost for robust fallback, enabling broad applicability with minimal added compute requirements.

### Limitations

The PROTAC-Splitter models demonstrate several notable limitations, which we discuss here. First and foremost, Transformer-based approaches occasionally generate small but chemically significant errors, such as extra atoms within ligands; in fact, it is usually the warhead that tends to have the most extra atoms (Table 3; on average 0.46 extra atoms on public data and 7.40 extra atoms on internal data), perhaps because there is most chemical variation in this substructure. While the fixing function corrects 16.89% of these cases in the synthetic held-out test set and 60.6% of these cases in the internal data set, challenging corner cases remain, particularly when errors span both linker attachment points, complicating automatic resolution.

Secondly, although the graph-based XGBoost model guarantees chemically valid outputs, its reliance solely on topological information limits its exact-match accuracy (42.20% on the synthetic held-out test set; 22.96% on the internal test set) and causes systematic misidentifications, particularly with macrocyclic or other types of complex PROTACs. We also observed that stereochemical mismatches at chiral centers account for a minor fraction of prediction errors ($\sim$1–2%; Appendix D.6). Future enhancements integrating 3D conformational awareness or stereochemical sampling approaches could mitigate these errors, though they are minor.

Ultimately, neither model can reliably distinguish non-PROTAC molecules, as it will split them anyways, incorrectly applying three-way splits for structurally unrelated compounds. This limitation underscores the need in future work for preliminary checks verifying the presence of PROTAC-like substructures, particularly if users are planning to push the limits of the model outside the applicability domain of PROTACs; for instance, molecular glues are occasionally misclassified as PROTACs and included in public datasets.

### Future work

Beyond immediate model refinements, our PROTAC-Splitter opens several promising avenues for future research. One direction involves investigating the additivity of physicochemical properties, such as cLogP or solubility, to determine whether they can be predicted or estimated from the properties of individual substructures. Such relationships could guide rational design strategies for new PROTACs. Another promising avenue is the integration of PROTAC-Splitter into generative AI frameworks, including RL models, to facilitate substructure-specific design objectives. The methodology could also be extended beyond PROTACs to other related heterobifunctional modalities, such as peptide-drug conjugates [21] or lysosome-targeting chimeras (LYTACs) [22], broadening its utility across drug discovery applications. Finally, incorporating 3D chemical information through distance geometry or equivariant neural networks could further enhance stereochemical precision and improve handling of structures with complex 2D graphical representations.

## Conclusion

Here we present the PROTAC-Splitter, a comprehensive framework for automated splitting of PROTAC molecules into their constituent ligands. Our contributions include the release of a large-scale synthetic PROTAC dataset (>1M structures) with carefully curated splits, the development of two complementary and easy-to-use models (Transformer- and XGBoost-based), and extensive benchmarking demonstrating robust performance in both public and internal datasets. Notably, we propose that a hybrid splitting approach would achieve complete coverage on novel datasets, effectively balancing accuracy and reliability. Despite limitations, PROTAC-Splitter represents a significant step toward scalable, automated annotation of PROTAC components, providing a robust foundation for advancing targeted protein degrader design and optimization.

## Supplementary Information

Supplementary Material 1.

Supplementary file (33 KB)

## Data Availability

Curated public data, the synthetic PROTACs dataset, and trained models are available for download at 10.5281/zenodo.15797310.

## Code availability

Code for training PROTAC-Splitter models, data curation, and synthetic PROTAC generation, is available at github.com/ribesstefano/PROTAC-Splitter.
