# Supplementary material for: PROTAC-Splitter: a machine learning framework for automated identification of PROTAC substructures
Source: J Cheminform. 2026 Feb 20;18:30. doi: 10.1186/s13321-025-01135-9 (PMC12924545; doi:10.1186/s13321-025-01135-9)
Supplement: Supplementary file 1 — Supplementary file (33 KB) [file 13321_2025_1135_MOESM1_ESM.pdf]

PROTAC-Splitter: A Machine Learning Framework for Automated Identification of PROTAC Substructures

Stefano Ribes[1], Ranxuan Zhang[1], Télio Cropsal[1], Anders Källberg[1], Christian Tyrchan[2], Eva Nittinger[2*], Rocío Mercado[1*]

[1]Department of Computer Science and Engineering, Chalmers University of Technology and University of Gothenburg, Chalmersplatsen 1, 412 96 Gothenburg, Sweden.
[2]Medicinal Chemistry, Research and Early Development, Respiratory and Immunology (R&I), BioPharmaceuticals R&D, AstraZeneca, Pepparedsleden 1, 431 83 Gothenburg, Sweden.

*Corresponding author(s). E-mail(s): eva.nittinger@astrazeneca.com; rocio.mercado@chalmers.se;

Appendix A Fixing Function

The fixing function forms the core of the Transformer-Δ wrapper in PROTAC-Splitter, repairing partially incorrect model outputs so that they exactly re-assemble into the target PROTAC. Given the canonical PROTAC SMILES and the model's prediction (*i.e.*, E3 ligase ligand, linker, and warhead SMILES), the routine applies a cascade of chemically aware checks and edits implemented in `RDKit`. It first attempts "light" fixes—swapping or relabelling attachment-point dummy atoms and, optionally, stripping stereochemistry—while verifying each attempt by assessing re-assembly. If these heuristics fail, it pinpoints the single erroneous fragment via systematic substructure deletion, reconstructs it by removing the mismatched part and replacing it with the correct fragment from the original PROTAC. A final safeguard flips ligand tetrahedral centres when a chirality mismatch is the sole obstacle to re-assembly. The procedure

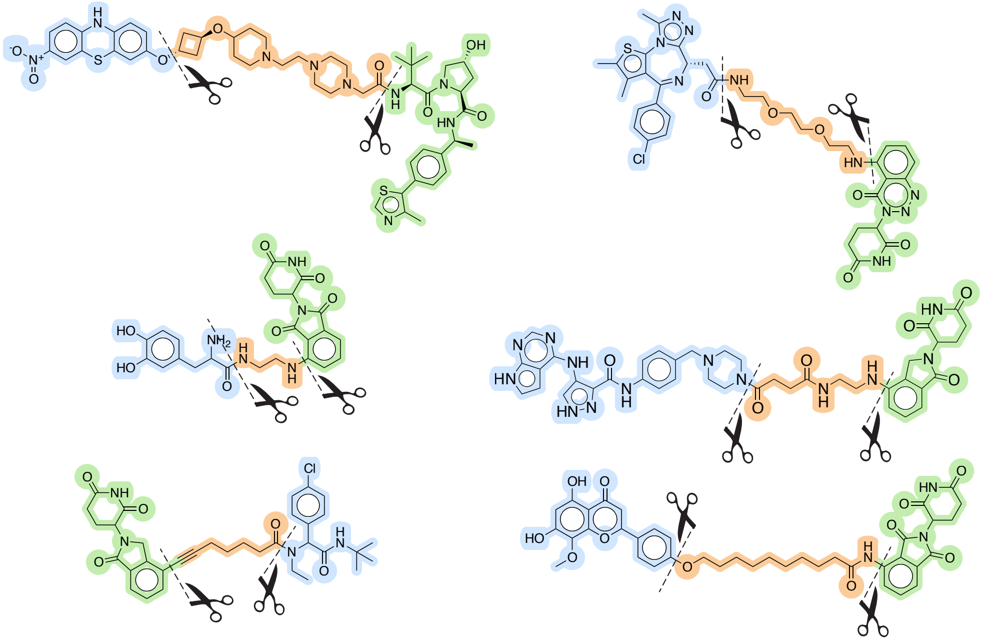

Fig. B1 Illustration of three manually-annotated PROTACs, on the left column, and computationally split, on the right column. Warhead substructures are highlighted in blue, linkers in orange, and E3 ligase ligands in green.

returns a chemically valid, fully matched triplet if fixing successes, raising overall reassembly accuracy of the Transformer predictions from 86% to 96% on public data and from 19% to 70% on structurally novel PROTACs.

Appendix B Examples of Split PROTACs

In Figure B1 we illustrate a few examples of PROTACs from the public data that could not be split by the algorithm, on the left column, and were manually annotated. In total, we manually annotated 103 structures. Additionally, the figure also shows, on the right column, three PROTACs automatically split by our data curation algorithm.

Appendix C Additional Details on the Synthetic Dataset

C.1 Morgan Fingerprints Parameters Selection

In Figure C2, we measure the number of collisions of Morgan fingerprints at different bit-width sizes and radius. The number of collisions is calculated by counting all compounds in the open held-out set that are mapped to the same fingerprint corresponding to (at least) another compound. As we can see, the fraction of compounds with colliding fingerprints is mainly driven by the selected radius, reaching less than 1% at radius = 8.

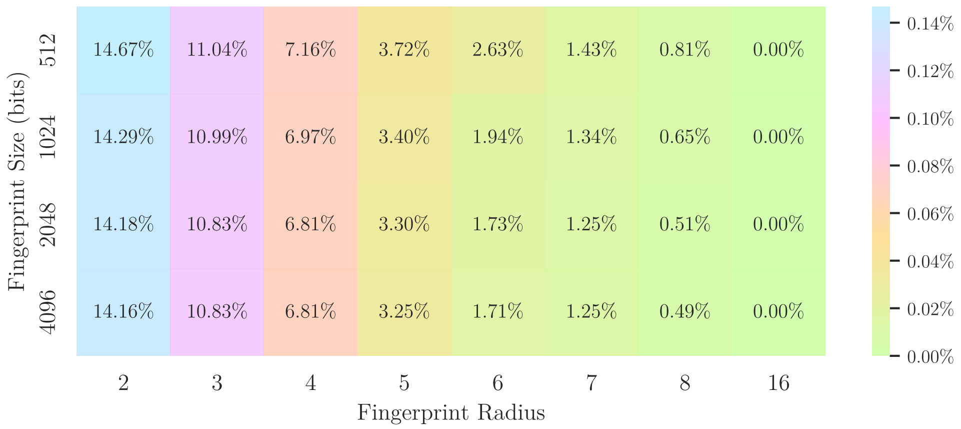

Fig. C2 Collision ratios at varying Morgan fingerprint bit widths and radii for the held-out public set. The percentages indicate the fraction of PROTACs that have fingerprint collisions with other PROTACs in the held-out public set using a given fingerprint width and radius.

C.2 Data Clustering and Splitting

To prevent information leakage while still training and evaluating the models on realistic chemical diversity, every ligand dictionary–$\mathscr{D}_{\text{WH}}$, $\mathscr{D}_{\text{E3L}}$, and $\mathscr{D}_{\text{LINK}}$–was partitioned into clusters to be assigned to either the training or the held-out splits. Ligands were encoded as 1 024-bit Morgan fingerprints (radius = 16, with chirality) and grouped with the Taylor-Butina algorithm. The similarity cut-offs were selected by jointly maximising the silhouette and Calinski-Harabasz scores while minimising the Davies-Bouldin index, giving thresholds of 0.82, 0.77, and 0.71 for E3 ligase ligands, linkers, and warheads, respectively. In particular, for each substructure type, to find a good similarity parameter, we computed the silhouette, Calinski-Harabasz, and Davies-Bouldin scores at different cut-offs, specifically from 0.5 to 0.98, with a 0.01 step size. We then plotted the scores against the cut-off parameters, and manually inferred a reasonable value, similarly to what done when analyzing a standard elbow plot. Figures C3, C4, and C5 show how the metrics change when clustering the warhead, linker, and E3 ligand substructures, respectively, at different cut-off similarity thresholds. The selected threshold values yielded 30 clusters for E3 ligase binders, 221 for linkers, and 230 for warheads. To guarantee genuine novelty in the evaluation sets we randomly withheld 11, 40, and 40 clusters of E3 ligase ligands, linkers, and warheads; as a result, 36 E3 ligase binders (14.2%), 300 linkers (15.6%), and 138 warheads (15.6%) never appear during training but are present in validation or test molecules, ensuring that many held-out PROTACs contain at least one previously unseen component.

C.3 Ligand Sampling

Once divided the ligands into seen and unseen during training, we proceeded to sample them to generate the synthetic PROTACs. The process is illustrated in Figure **??**c and

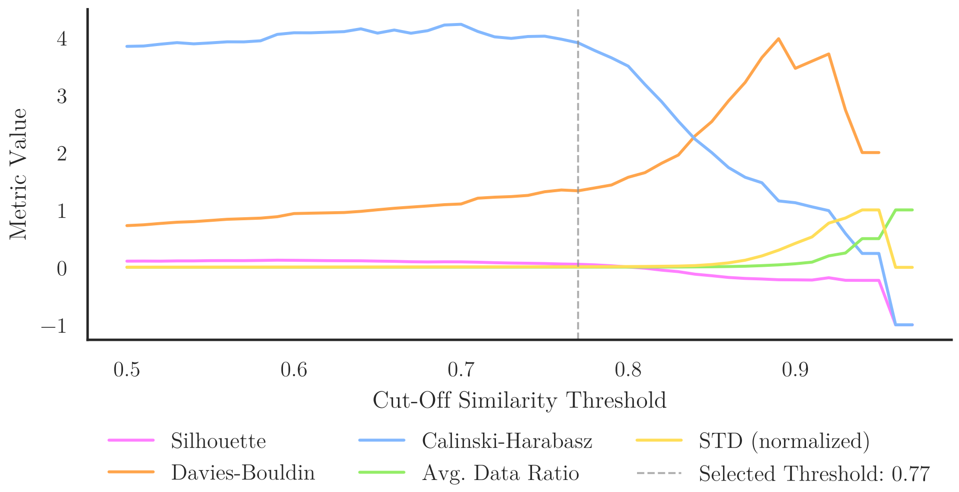

Fig. C3 Clustering metrics (silhouette, Davies-Bouldin, and Calinski-Harabasz scores) against increasing similarity cut-off thresholds for warhead substructures. The additional metrics, "Avg. Data Ratio" and "STD", represent the cluster size average and standard deviation compared to the available data.

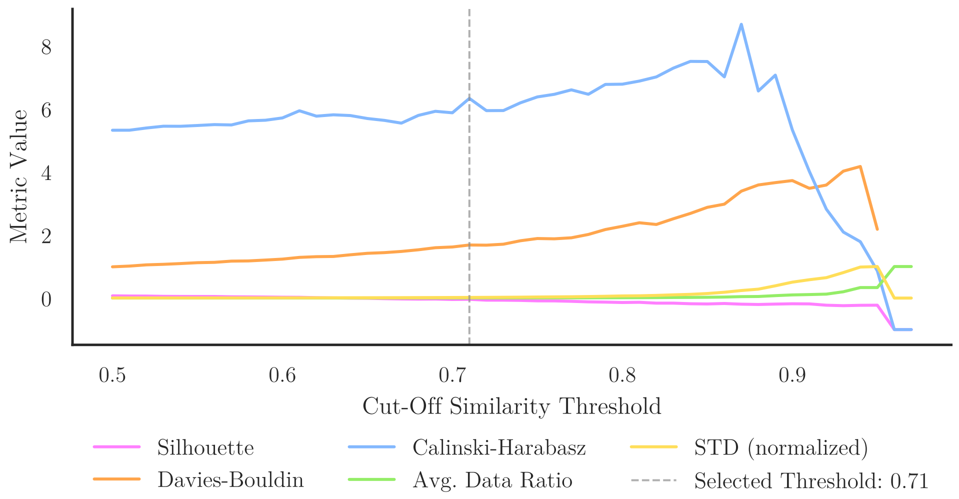

Fig. C4 Clustering metrics (silhouette, Davies-Bouldin, and Calinski-Harabasz scores) against increasing similarity cut-off thresholds for linked substructures. The additional metrics, "Avg. Data Ratio" and "STD", represent the cluster size average and standard deviation compared to the available data.

described in detail below. When sampling, we want to reproduce the connection type distribution at the ligands attachment points of the curated ("real") PROTACs. To do so, we first extracted and counted the SMARTS representation of the atoms around

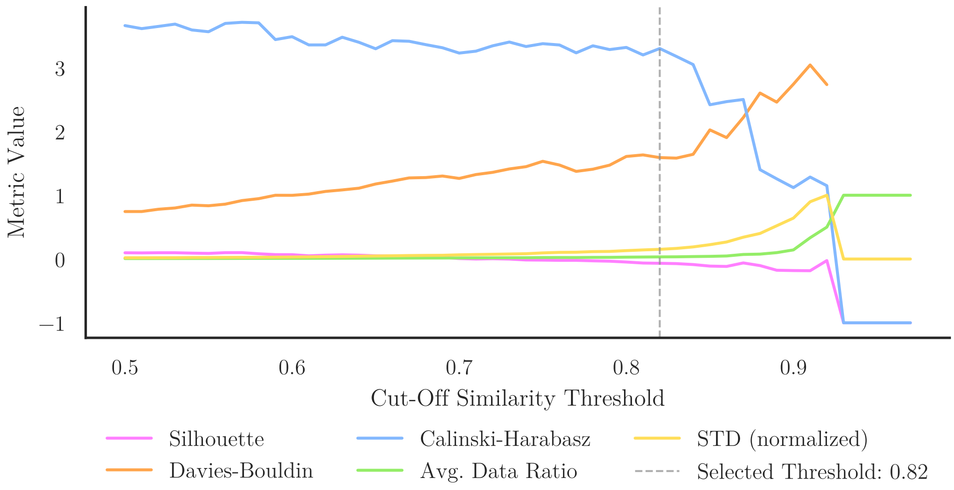

Fig. C5 Clustering metrics (silhouette, Davies-Bouldin, and Calinski-Harabasz scores) against increasing similarity cut-off thresholds for E3 ligase ligand substructures. The additional metrics, "Avg. Data Ratio" and "STD", represent the cluster size average and standard deviation compared to the available data.

the attachment points. We included hydrogen atoms and all atoms within two atoms distance from the attachment point. Next, we begin by sampling connection types for both warhead and E3 ligase components according to their respective probability distributions. These connection types serve as keys to retrieve a set of compatible ligands molecules associated to the sampled connection types. For each warhead-E3 ligase binder pair, we then identify a set of compatible linker molecules by finding the intersection of linkers that can connect to both connection types. A warhead, an E3 ligase binder, and a linker are then uniformly sampled from the respective sets of compatible ligands. The generation algorithm then constructs candidate PROTAC molecules by joining the three components together, attempting to merge them into a single molecule using `RDKit`'s `molzip` function. Finally, only molecules that pass all validation steps, including sanitization checks to ensure chemical validity, and re-assembly verification, are retained. Furthermore, we discard any generated PROTAC that is equal to the "real" existing PROTACs and make sure that generated PROTACs do not leak across train, validation, and test sets.

C.3.1 Connection Type Distributions

In Table C1 we summarize the top connection types present in the public PROTACs curated from PROTAC-DB and PROTAC-Pedia. These distributions were then sampled from in order to simulate similar distributions of connection types in the synthetic PROTAC dataset constructed in this work.

Table C1 Top-10 most common connection types (SMARTS patterns) for warhead-linker bonds and linker-E3 ligase binder bonds. The table also reports the number of open source ("real") PROTACs containing each specific connection type, as well as the percentage of the PROTACs in the public set that this accounts for.

Connection Type	Percentage	Count
**Warhead-Linker**		
[H]-[N](-[C])-[C](-[C])=[O]	25.70%	1,457
[C]-[C](=[O])-[N](-[C])-[C]	14.50%	822
[H]-[C](-[H])(-[C])-[N](-[C])-[C]	8.32%	472
[C]:[C](:[C])-[O]-[C]	7.14%	405
[H]-[C](-[H])(-[C])-[C](-[H])(-[H])-[N]	5.17%	293
[C]:[C](:[C])-[N](-[C])-[C]	5.08%	288
[H]-[C](-[H])(-[C])-[O]-[C]	3.14%	178
[H]-[N](-[O])-[C](-[C])=[O]	2.56%	145
[H]-[N](-[N])-[C](-[C])=[O]	2.54%	144
[C]#[C]-[C](:[C]):[C]	2.47%	140
**Linker-E3 Ligase Ligand**		
[H]-[N](-[C])-[C](-[C])=[O]	45.13%	2,559
[H]-[N](-[C])-[C](:[C]):[C]	23.47%	1,331
[C]:[C](:[C])-[O]-[C]	12.06%	684
[H]-[N](-[C])-[C](-[H])(-[H])-[C]	4.97%	282
[C]#[C]-[C](:[C]):[C]	3.54%	200
[C]:[C](:[C])-[N](-[C])-[C]	3.02%	171
[H]-[C](-[H])(-[C])-[C](:[C]):[C]	1.20%	68
[C]-[C](=[O])-[N](-[C])-[C]	1.20%	68
[H]-[C](-[H])(-[O])-[C](-[H])(-[H])-[O]	1.13%	64
[H]-[C](-[H])(-[C])-[O]-[C]	0.53%	30

Appendix D Additional Transformer-PROTAC-Splitter Results

D.1 Hyperparameter Tuning

The hyperparameter optimization for the Transformer-PROTAC-Splitter model is conducted using a systematic search with Optuna, integrated into the Hugging Face training framework. The tuning process primarily focuses on the learning rate and the proportion of warmup steps used in the learning rate schedule. Depending on the chosen learning rate scheduler, among cosine decay, cosine decay with restarts, or plateau-based reduction, additional scheduler-specific parameters are also optimized, including the number of decay cycles or the minimum allowable learning rate. To prevent wasting resources on unpromising configurations, we employ an early stopping mechanism that combines median-based pruning with a custom strategy that monitors progress over time and halts training if the performance stagnates beyond a predefined

patience window, provided a minimum improvement threshold is not met. The objective function is setup to maximize exact-match accuracy. The hyperparameter search leverages low-discrepancy sampling techniques to systematically explore the search space and identify the configuration that yields the best empirical performance. For each of the three learning rate schedulers mentioned above, we spawn 25 trials.

D.2 Data Efficiency

Figure D6 summarizes the data-efficiency study for the Transformer-PROTAC-Splitter. As the fraction of synthetic training data grows from 25% to 100%, validity remains consistently high (97%), while reassembly accuracy rises only modestly from 77% to 79%. Exact-match accuracy shows a similar, gently increasing trend, improving by just 2.5 percentage points overall. The magenta trace reports "Exact-match (normalized)", defined here as the absolute heavy-atom difference between the reassembled prediction and the input PROTAC divided by the number of heavy atoms in the PROTAC molecule; it therefore quantifies the fraction of atoms erroneously added or removed. Importantly, this normalized error stays below 4% across all training sizes, confirming that even smaller training sets introduce only minor performance deviations. Together, these results indicate diminishing returns beyond roughly half of the available training set, suggesting that the current 1.3 M-molecule corpus already captures most learnable splitting patterns for the task.

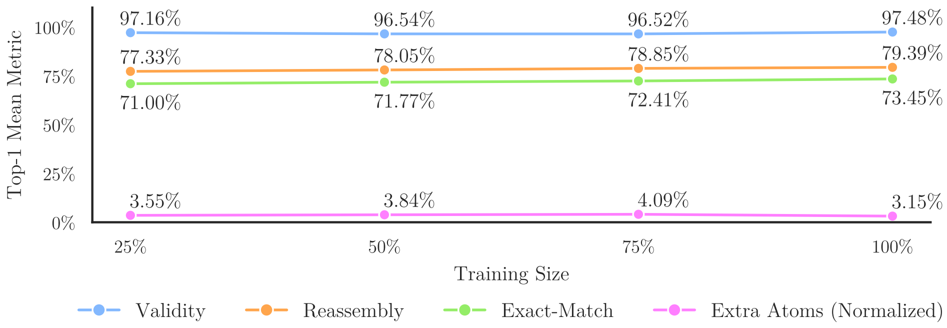

Fig. D6 Performance metrics for the PROTAC-Splitter-Transformer trained on 25%, 50%, 75%, and 100% of the synthetic training data.

We also computed F1-score, precision, recall, and accuracy of the XGBoost-PROTAC-Splitter in classifying line node edges for splitting. The metrics were collected at different ratios of the training set, specifically at 0.1%, 0.5%, 1%, and 2.5%, as reported in Figure D7. Despite the slight increase in the recall score, all other metrics settle quickly and become constant as we increase the amount of training data, suggesting a negligible gain in adding more training samples. One possible explanation for this trend might be the "similarity" of the bonds to split. For example, a popular splitting point is represented by amide bonds: despite the model seeing different molecular graphs, and so different features, edges corresponding to amide bonds will

all have very similar chemical characteristics, like the number and atom types of the neighboring nodes.

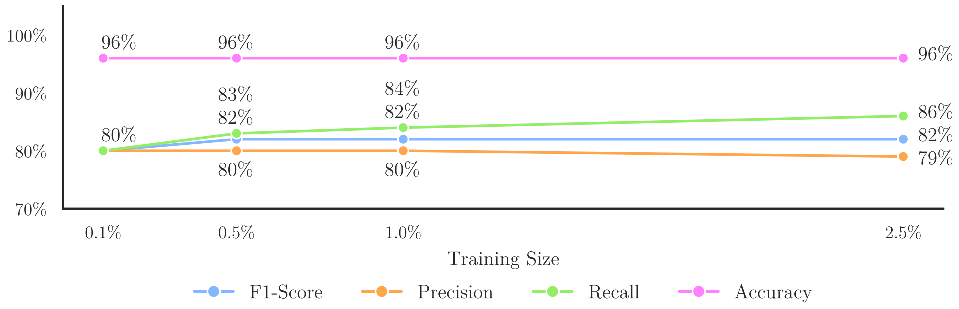

Fig. D7 Performance metrics for the XGBoost model trained on 0.1%, 0.5%, 1%, and 2.5% of the synthetic training data.

D.3 Effect of SMILES Augmentation

Table D2 summarizes the performance of the Transformer-PROTAC-Splitter when trained with and without SMILES randomization of 15% of the training data. All metrics improved consistently when SMILES augmentation was introduced, leading us to apply the augmentation in all our experiments.

Table D2 Effect of SMILES randomization on the performance of the held-out open dataset for the Transformer and Transformer-Δ models.

Metric	No SMILES Rand.	Rand. SMILES	No SMILES Rand. (Δ)	Rand. SMILES (Δ)
Validity	95.50%	97.48%	99.10%	99.52%
Reassembly	78.86%	79.39%	97.42%	97.83%
Exact-match	72.35%	73.45%	85.67%	87.40%
Extra atoms	3.24	2.06	0.67	0.45

D.4 Comparing Generation Strategies

We evaluated six decoding strategies for sequence generation in the Transformer model: greedy search, multinomial sampling, beam search, diverse beam search, beam search with multinomial sampling, and contrastive decoding. For all beam-based strategies, we used a fixed beam width of 5.

These decoding strategies were compared specifically for proposing PROTAC splits, using four metrics: validity, re-assembly, heavy atoms difference, and exact matching. Metric scores were calculated for predicted PROTAC SMILES and averaged across 5,670 samples from the held-out, open-source set.

Beam search decoding achieved the highest performance and was thus selected for subsequent evaluations of the Transformer-based PROTAC-Splitter. A comprehensive

summary of the top-5 performance of all strategies on the open held-out set is presented in Table D3.

Table D3 Comparison of different sampling strategies for the Transformer-PROTAC-Splitter.

Metric	Beam Search	Multinomial Sampling	Diverse Beam Search	Greedy	Contrastive Search
Validity	97.46%	96.51%	96.51%	97.48%	96.49%
Reassembly	79.39%	79.39%	79.39%	79.39%	79.41%
Exact-match	73.45%	73.48%	73.48%	73.45%	73.57%
Extra atoms	2.06	2.70	2.70	2.06	2.71

D.5 Top-5 Performance for Transformer-PROTAC-Splitter

For the Transformer-PROTAC-Splitter we report in Tables D4 and D5 the top-N scores for every single PROTAC and its substructures, calculated as follows: given the N predictions SMILES generated after a beam search, we first filter those that successfully re-assemble into the input PROTAC, we then select and score the one with the highest likelihood. We also show the performance of the model when applying the fixing function before filtering unsuccessful predictions.

Compared to the top-1 scores in Tables **??**, top-5 performance on public data improves reassembly by 2.76% and exact matching by 1.81% absolute percentage points. On AstraZeneca's internal data the improvement is minor, 0.76% for reassembly and 0.48% for exact matching. Regarding Transformer-Δ performance, considering top-5 predictions yields negligible improvements. A similar trend is observed in Table D5. Overall, applying the fixing function to top-1 predictions shadows the benefit of filtering predictions that successfully re-assemble.

D.6 Performance Ignoring Stereochemistry

We report in Table D6 the reassembly score of the PROTAC-Splitter models when removing stereochemistry from the input PROTACs, its labels, and the model predictions.

Appendix E Heuristic-PROTAC-Splitter

While our main models learn splitting rules from data, we developed a rule-based reference that works without probabilistic models nor training. Heuristic-PROTAC-Splitter offers a training-free baseline that looks for two bonds whose removal isolates the linker: intuitively, linker atoms often sit on narrow "bridges" that connect two bulky domains (*i.e.*, the E3 ligase binders and the warhead); by ranking atoms according to how many shortest paths in the molecular graph pass through them, the algorithm pinpoints where a cut is most likely to separate the three PROTAC components.

The procedure begins with evaluating the betweenness centrality for every atom in the graph representation of the molecule. Ring atoms are ignored, and only those that are bridges in the graph are considered further. The two highest-scoring bridge atoms (usually at the "center" of the linker) seed a candidate set, which is expanded with

Table D4 Top-5 evaluation metrics on public and internal datasets using the Transformer-PROTAC-Splitter model. The 'Top-5-Δ' column reports scores when applying the fixing function to the 5 predictions. The number of extra atoms should be close to zero, whereas a higher value in the other metrics is preferred.

Dataset (Size)	Metric	Top-5	Top-5-Δ
Public (5,670)	Validity	97.48%	99.51%
	Reassembly	82.15%	96.44%
	Exact-match	75.26%	85.80%
	Extra atoms	2.03	0.46
Internal (2,256)	Validity	73.54%	88.92%
	Reassembly	10.51%	70.35%
	Exact-match	4.74%	18.71%
	Extra atoms	17.96	7.70

Table D5 Top-5 evaluation metrics for the substructures of the public and internal datasets using the Transformer-PROTAC-Splitter model. The 'Top-5-Δ' column shows the score after applying the fixing function. The number of extra atoms should be close to zero, whereas a higher value in the other metrics is preferred.

Dataset (Size)	Substructure	Metric	Top-5	Top-5-Δ
Public (5,670)	Warhead	Validity	97.97%	99.59%
		Exact-match	85.64%	90.88%
		Extra atoms	0.44	0.01
	Linker	Validity	99.79%	99.95%
		Exact-match	84.54%	87.65%
		Extra atoms	0.23	0.09
	E3 Ligand	Validity	99.70%	99.95%
		Exact-match	89.45%	91.74%
		Extra atoms	0.06	0.06
Internal (2,256)	Warhead	Validity	73.98%	89.36%
		Exact-match	11.92%	36.61%
		Extra atoms	7.39	3.59
	Linker	Validity	98.94%	98.94%
		Exact-match	18.26%	18.97%
		Extra atoms	-0.73	-0.99
	E3 Ligand	Validity	99.20%	99.20%
		Exact-match	40.25%	42.86%
		Extra atoms	0.84	0.78

any non-ring neighboring atoms that either exceed a user-defined centrality threshold τ (0.4 by default) or are enclosed by the current set, *e.g.*, the atoms of a ring structure within a linker. This small cluster typically outlines the linker region.

Table D6 Top-1 reassembly metric on the public and internal datasets for both the XGBoost- and Transformer-based PROTAC-Splitter models when we neglect stereochemistry information. The "Transformer-Δ" column reports Transformer performance after applying the fixing wrapper function. A higher number indicates better performance. Both PROTACs and corresponding labels have been strip from stereochemistry information before being processed by the models.

Dataset (Size)	Metric	XGBoost	Transformer	Transformer-Δ
Public (5,670)	Reassembly	**100%**	80.96%	97.76%
Internal (2,256)	Reassembly	**100%**	11.35%	70.35%

The next step is to check whether removing the candidate linker atoms from the PROTAC structure results in more than three fragments. This might happen, for example, when a linker includes atoms with low centrality that "branch out" from the center of a linear alkyl chain. To account for this scenario, all bonds that connect the candidate set to the rest of the molecule are enumerated pairwise. Each pair is fragmented with `RDKit`; pairs that do not yield exactly three fragments are discarded. The remaining pairs are ranked by the balance of fragment sizes—the lower the standard deviation, the higher the rank—and the top pair is chosen as the predicted cut sites.

Finally, the algorithm labels the three fragments: the fragment containing two attachment points is the linker; of the other two, the one closest, by average Tanimoto similarity on 1,024-bit Morgan fingerprints (radius = 16), to a set of representative E3 ligase ligands is labeled as the E3 ligase binder; the remaining fragment is the warhead. Dummy atoms at attachment points are then standardized so that the E3 ligase-facing attachment is `[:2]` and the warhead-facing attachment is `[:1]`, ensuring that the predicted triplet reassembles exactly into the parent PROTAC.
